# Heterogeneity in the in vitro susceptibility of *Loa loa* microfilariae to drugs commonly used in parasitological infections

**DOI:** 10.1186/s13071-018-2799-3

**Published:** 2018-04-04

**Authors:** Abdel J. Njouendou, Fanny F. Fombad, Maeghan O’Neill, Denis Zofou, Chuck Nutting, Patrick C. Ndongmo, Arnaud J. Kengne-Ouafo, Timothy G. Geary, Charles D. Mackenzie, Samuel Wanji

**Affiliations:** 1 Parasites and Vectors Biology Research Unit (PAVBRU), Department of Microbiology and Parasitology, Faculty of Science, University of Buea, Buea, Cameroon; 2 Institute of Parasitology, McGill University, Ste-Anne-de-Bellevue, QC H9X 3V9 Canada; 3 Biotechnology unit, Department of Biochemistry and Molecular Biology, Faculty of Science, University of Buea, Buea, Cameroon; 4 Department of Biological Sciences, Western Michigan University, Kalamazoo, MI 49008 USA; 5 Department of Pathobiology and Diagnostic Investigation, Michigan State University, East Lansing, MI 48824 USA; 6 Filariasis Programmes Support Unit, Liverpool School of Tropical Medicine, Pembroke Place, Liverpool, L3 5QA UK

**Keywords:** Antimalarial, Anthelmintics, Trypanocides, Imatinib, *Loa loa* microfilariae

## Abstract

**Background:**

Co-infection with loiasis remains a potential problem in control programs targeting filarial infections. The effects of many anti-parasitic drugs often administered to *Loa loa* infected people are not well documented. This study compared the in vitro activity of several of these drugs on the viability of *L. loa* microfilariae (mf).

**Methods:**

Human strain *L. loa* mf were isolated from baboon blood using iso-osmotic Percoll gradient, and cultured in RPMI 1640/10% FBS with antimalarial drugs (mefloquine, amodiaquine, artesunate, chloroquine and quinine), anthelmintics (ivermectin, praziquantel, flubendazole and its reduced and hydrolyzed metabolites), two potential trypanocidal agents (fexinidazole and Scynexis-7158) and the anticancer drug imatinib. The drug concentrations used varied between 0.156 μg/ml and 10 μg/ml. Mf motility (CR_50_ = 50% immotility) and a metabolic viability assay (MTT) were used to assess the effects of these drugs on the parasites.

**Results:**

Mf in control cultures showed only a slight reduction in motility after 5 days of culture. Active inhibition of *Loa loa* motility was seen with mefloquine and amodiaquine (CR_50_ values of 3.87 and 4.05 μg/ml, respectively), immobilizing > 90% mf within the first 24 hours: mefloquine killed the mf after 24 hours of culture at concentrations ≥ 5 μg/ml. SCYX-7158 also induced a concentration-dependent reduction in mf motility, with > 50% reduction in mf motility seen after 5 days at 10 μg/ml. The anticancer drug imatinib reduced mf motility at 10 μg/ml from the first day of incubation to 55% by day 5, and the reduction in motility was concentration-dependent. Praziquantel and fexinidazole were inactive, and FLBZ and its metabolites, as well as ivermectin at concentrations > 5 μg/ml, had very minimal effects on mf motility over the first 4 days of culture.

**Conclusions:**

The considerable action of the anti-malarial drugs mefloquine and amodiaquine on *Loa* mf in vitro highlights the possibility of repurposing the existing anti-infectious agents for the development of drugs against loiasis. The heterogeneity in the activity of anti-parasitic agents on *Loa loa* mf supports the need for further investigation using animal models of loiasis.

**Electronic supplementary material:**

The online version of this article (10.1186/s13071-018-2799-3) contains supplementary material, which is available to authorized users.

## Background

Loiasis, a neglected tropical disease also known as African eye worm, is caused by the nematode *Loa loa*, transmitted through the bite of deer flies (*Chrysops* spp.). Loiasis is thought to cause relatively little pathology except for localized mild to moderate pruritus and oedema, and distress due to the occasional sub-conjunctival migration of the adult worm. Although the disease is endemic in Central and West Africa [1], 101 cases were reported outside Africa in the past 25 years (1986–2011), of which 61 (60.4%) were in Europe and 31 (30.7%) in the USA [2]; however, all these patients had spent time in an African endemic area.

Although relatively asymptomatic when untreated, *L. loa*-infected people, when administered the anti-parasitic agents commonly used for other filarial infections such as onchocerciasis and lymphatic filariasis, may experience severe adverse effects, especially in people carrying very high loads of circulating *L. loa* mf (> 8000 mf/ml) treated with diethylcarbamazine or ivermectin [3–5]. This post-treatment phenomenon has been an impediment to programmes administering ivermectin to eliminate onchocerciasis and lymphatic filariasis, both of which can co-exist with loiasis [4, 5]. Fatal complications involving encephalopathy have been documented during the treatment of loiasis patients [3, 6, 7]. Further, the cross-reactivity of the Immuno-chromatographic card (ICT) with loiasis [8, 9] has impaired the mapping of lymphatic filariasis in the rainforest block of central Africa, where loiasis is endemic.

No registered safe drug is available for the control of loiasis and no vaccine is available for loiasis or other filarial infections, despite the considerable efforts made to understand the mechanisms and major effectors of the immune response induced by the parasite in the host [10, 11]. Pending the development of such a vaccine, the development of a drug that can safely clear the infection would be highly valuable for control and elimination programmes.

In the search for a safe drug for use in loiasis regions, there is a need of an in vitro system to study the effects of candidate drugs on the parasite viability. Although there has been considerable success in developing protocols to maintain these parasites in primates [7, 12, 13], culture systems more amenable for higher-throughput drug testing remain unavailable. Baboons, along with mandrills, are important models for the study of *L. loa* since they, like humans, are susceptible to infection and develop prolonged microfilaraemia without developing clinical disease [7, 13]. Despite the success of maintaining *L. loa* parasites in baboons, this model is practically inappropriate for in vivo drug screening purposes at large scales; the numbers required and cost of animal maintenance, along with ethical and environmental constraints, prevent the routine use of these protected animals for research.

The development of an in vitro system for drug testing against this parasite has been impeded by the unavailability of the parasites in most laboratory settings, even though blood from infected humans has been used in prior screening of plant extracts [14]. Nevertheless, this source of parasites is ethically inappropriate. However, it has been shown that baboons inoculated with third-stage larvae of a human strain of *L. loa* developed patent infections after 135 to 148 days; in each animal which became patent, microfilaremia rose rapidly to high levels, followed by a suppression of microfilaremia during the 4th month of patency. After splenectomy, microfilariae reappeared in the peripheral blood in large numbers [13]. Thus, splenectomised baboons are a good animal reservoir of *L. loa* mf. In this study, this source of parasite material was used to investigate the susceptibility of mf to L3 antiparasitic drugs.

The study described here addresses the in vitro activity of antiparasitic drugs on *L. loa* mf in culture. Ivermectin, an obvious control for this study, is the drug of choice for the treatment of onchocerciasis. This semi-synthetic macrolide is effective in vivo against mf (but not adult worms) of *L. loa*. It shows little in vitro activity against mf [15], and this is thought to be related to the requirement of the host immune system to mediate mf killing by ivermectin in vivo [16]. The anticancer imatinib (Gleevec; GLV) was used because it was recently added to the list of filaricidal drugs [17] and its microfilaricidal property was reported against *Brugia* [18]. The benzimidazole flubendazole (FLBZ) has been suggested as a candidate macrofilaricide for onchocerciasis [19], and has been extensively investigated in a range of animal models since its discovery over 40 years ago. The benzimidazoles are broad spectrum anthelmintics that bind preferentially to parasite β-tubulin leading to the disruption of microtubules and, ultimately, to the death of the parasite. FLBZ exhibits macrofilaricidal activity against *Brugia pahangi* [20, 21], *Breinlia booliati* [22], and, as a pro-drug of FLBZ (UMF-078), on *Onchocerca ochengi* [23]. In addition, several studies in other filarial species have indicated that FLBZ lacks or has only very little activity against mf stages. For example, it is ineffective against *B. pahangi* mf [21], and shows very low activity compared to ivermectin against mf in a mouse model of *Onchocerca lienalis* [24]. It was recently shown to inhibit the development of *B. malayi* mf to L3 in *Aedes aegypti*, while it did not affect the viability of *L. loa* and *B. malayi* mf at 10 μM after 72 h exposure in vitro [25]. Praziquantel and the antimalarial drugs mefloquine, artesunate, chloroquine and amodiaquine were tested as they exhibit anthelmintic activity against *Schistosoma* species [26–30]. Fexinidazole and Scynexis-7158 are new clinical candidates currently under development against sleeping sickness. Since the endemicity of human African trypanosomiasis (HAT) overlaps with that of loiasis in some area of sub-Saharan Africa [1, 31], there is a need to predict the effect of these drugs on the viability of *L. loa* mf. This will help to prevent fatal complications such as those that have been observed post-mass treatment of onchocerciasis with ivermectin in loiasis co-endemic areas. These two new oral candidates were suggested by the Drug for Neglected Disease initiative (DNDi) and partners for clinical trial studies. Fexinidazole (5-nitro-imidazole), was successfully evaluated in Phase I between 2009 and 2012 and its potential as an oral, safe and well-tolerated drug was demonstrated [32, 33]. Phase II/III trials were launched in December 2012 in Democratic Republic of the Congo (DRC) followed by Central African Republic and was completed in November 2017. The oxaborole Scynexis-7158 (SCYX-7158) entered the phase 1 clinical trials in March 2012 in Paris, and that was successfully completed by 2015. These candidates were selected on the basis of their safety and their potential to treat advanced-stage sleeping sickness as observed in preclinical studies [34–36].

## Methods

### Source of parasites

*Loa loa* mf were obtained from baboons (*Papio anubis*) kept in captivity and infected with the human strain of *L. loa* according to a modified protocol of Orihel et al. [12, 13] and Wanji et al. [37]. Briefly, the animals were splenectomized and infected by subcutaneous injection of 100 infective larvae (L3) of *L. loa* from wild-caught *Chrysops* flies, with the animals being carefully monitored clinically and parasitologically until patency [38].

Baboons were acquired from hunters in different regions of Cameroon and maintained at a refurbished monkey house in Kumba. They were fed fruits, fats and proteins supplemented with vitamins.

### Culture constituents and test agents

Complete culture medium (CCM) used to maintain the parasites in vitro was RPMI 1640 supplemented with 2 g/l sodium bicarbonate, 25 mM Hepes, 10% fetal bovine serum (FBS), 100 U penicillin, 100 μg/ml streptomycin and 2.5 μg/ml amphotericin B. Stock-isotonic Percoll (SIP - Pharmacia, Uppsala, Sweden) was prepared by mixing 1 part 10× concentrated RPMI 1640 and 9 parts Percoll; dilutions were made in RPMI 1640. DMSO, RPMI 1640, Hepes, sodium bicarbonate, FBS and amphotericin B were purchased from Sigma Aldrich (St Louis, MO, USA) while penicillin-streptomycin solutions (Pen-Strep) were obtained from Gibco/Thermo Fisher (Grand Island, NY, USA). All chemicals were culture grade.

FLBZ and its principal metabolites, reduced flubendazole (R-FLBZ) and hydrolyzed flubendazole (H-FLBZ), were obtained from Epichem Pty Ltd, Murdoch University Campus, South Street, Murdoch WA 6150, Australia. Imatinib was a gift from T. Nutman, NIH, Bethesda, MD, USA. Ivermectin (IVM), Praziquantel (PZQ), mefloquine (MFQ), artesunate (ATS), quinine (QN), chloroquine (CQ) and amodiaquine (AQ) were purchased from Sigma-Aldrich (St Louis, MO, USA). Fexinidazole and SCYX-7158 were provided by DNDi.

### Preparation of parasites

Blood was aseptically collected from infected baboons by venipuncture into EDTA vacuum tubes. Mf extraction began immediately upon arrival in the laboratory using a modified iso-osmotic Percoll extraction method [39, 40]. Discontinuous gradient solutions containing 40%, 50% and 65% SIP were prepared in 15 ml centrifuge tubes. An aliquot of 2 ml of undiluted blood was pipetted and layered gently onto the gradient and the tubes centrifuged at 900× *g* for 10 min at 25 °C. The layer containing mf was removed with a syringe and filtered gently through a 5-μm pore cellulose filter. The filter was immediately transferred to a Petri dish containing CCM and incubated at 37 °C for 5 min. After removing the filter, the remaining fluid containing the mf was centrifuged (300× *g* for 10 min, 25 °C), the mf quantified and transferred to culture plates. The quality of mf and the culture conditions were initially assessed by culturing the first batch of parasites for 40 days. During this pre-optimization step, mf retained optimal motility for at least 10 days, confirming that during the 5 days of screening, there would be minimal risk of mf dying due to the stress of the culture conditions. Immediately after purification, all batches were free of dead parasites, and the proportion of sluggish mf was usually below 1/100.

### Preparation of drug solutions

Stock solutions of each drug were prepared by dissolving 5 mg in 500 μl DMSO in an Eppendorf tube which was then vortexed. For AQ, CQ and GLV, the DMSO component was replaced by double de-ionized water. Following this, 20 μl of each suspension was transferred into 10 ml CCM, then two-fold dilutions were made to obtain final concentrations between 0.156–10 μg/ml. For the control, 20 μl DMSO was mixed with 10 ml CCM to make a 0.2% solution. The final concentration of DMSO was 0.1% in all wells including the negative controls.

### Cultures

**Fig. 1:**
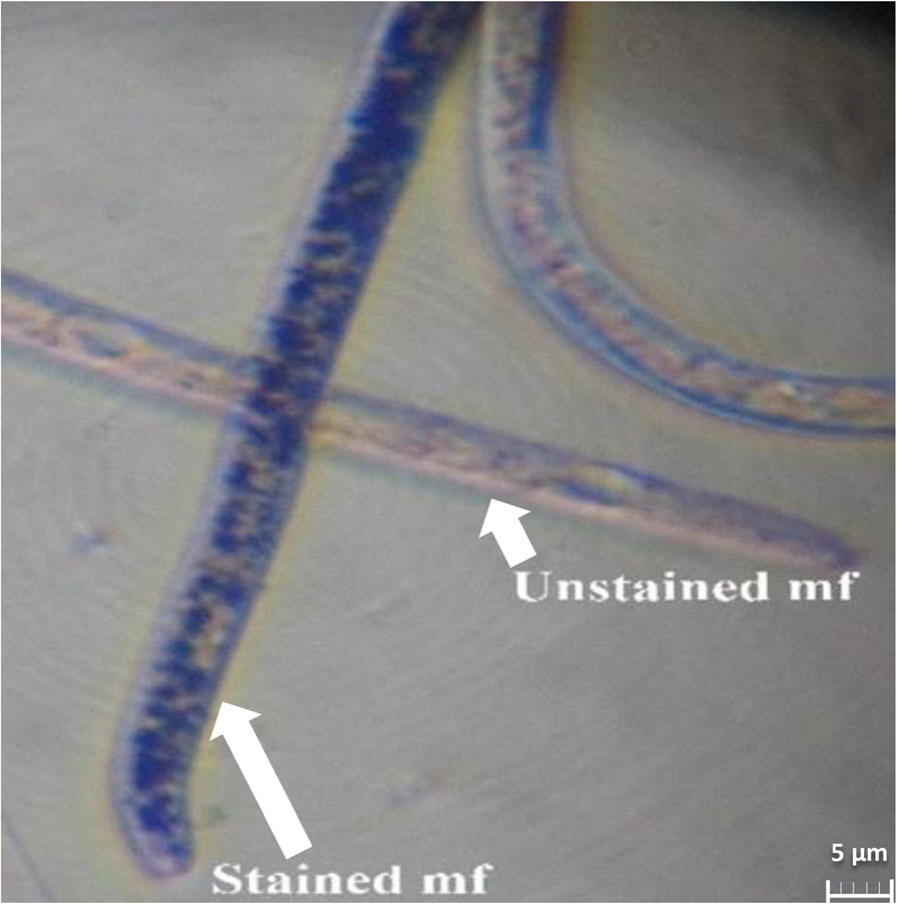
Photomicrograph (×400) of stained and unstained *L. loa* microfilariae after incubation with MTT reagent. This micrograph shows a live *L. loa* mf that metabolized MTT into blue formazan (left arrow) and a dead mf that is unstained (right arrow)

Parasites were cultured in 48-well plates. Each well contained 200 μl drug solution at the desired concentration and 200 μl parasite suspension containing 50 ± 5 mf. For the control wells, 200 μl DMSO (0.2%) in CCM or CCM only were added to 200 μl parasite suspension containing approximately 50 mf. Four replicates were tested for each drug concentration and each experiment was repeated twice. Motility was assessed under an inverted microscope at 24 h intervals for 5 days. The mf motility was scored on a scale of: 0 (immotile), 1 (intermittent shaking of the head and/or tail region), 2 (sluggish and motile), 3 (highly active and motile) [41]. On day 5, parasites with no movement were further assessed for viability by measuring the metabolic reduction of 3-(4, 5-dimethylthiazol-2-yl)-2, 5-diphenyltetrazolium bromide (MTT) to formazan [42]. During this process, mf were incubated for 1 h in a 48-well plate containing 40 μl/well MTT (0.5 mg/ml) in phosphate-buffered saline (PBS) and observed under an inverted microscope for color change in the parasite body [43]; live parasites stained blue while dead worms were unstained (Fig. 1).

### Data processing

Cultures were evaluated on a relative scale. After scoring motility daily from day 0 of culture to the last day of each experiment by counting the number (Ni) of parasites with the same score (Si), motility (%) of parasites in each well at each time point was summarized using the following formula:

$$ \mathrm{Motility}\ \left(\%\right)=\frac{\sum \left(\mathrm{N}i\times \mathrm{S}i\right)}{3\times \sum \mathrm{S}i}\times 100 $$

Computed motility ranged from 0% if all mf in the well were immotile (score 0) to 100% when all worms are actively motile (score 3, the maximum). This formula was built based on the evaluation of relative score described for an in vitro screening assay using albendazole, diethylcarbamazine and IVM against *Brugia malayi* infective larvae [44]. In the formula, the value 3 represents the highest score.

Percent reduction in motility for each drug concentration was calculated on days 3 and 5 using the following formula.

$$ \%\mathrm{reduction}=\frac{\mathrm{Control}-\mathrm{drug}}{\mathrm{Control}}\times 100 $$

The extent of mf mortality was reported as % immotile (score 0) worms. Raw data were saved on a spreadsheet in Microsoft Excel 2013, and the motility response generated. Graphical displays were generated using GraphPad Prism software. Data were exported to SPSS version 20 for statistical analysis. Spearman’s rank correlation rho was used to assess the association between the drug concentration and the reduction in mf motility on day 5. The effect of drugs on the motility of parasites was also appreciated by estimating the concentration required for each drug to reduce the motility of the mf by 50%, defined as CR_50_. The lower the value of CR_50_, the higher the activity of the drug. The CR_50_ was estimated using GraphPad Prism software. Results of replicates were expressed as mean ± standard deviation (SD). The value of this parameter was used to compare the effects of the drugs on mf. The effects of drug concentrations on motility were compared using non-parametric tests. The Kruskal-Wallis one-way analysis test was used to assess the global significant differences between the median CR_50_ of the active drugs and when a difference was detected, Dunn’s *post-hoc* test was applied for pairwise multiple comparisons of the ranked data. This analysis was performed using the Pairwise Multiple Comparisons of Mean Rank Sums (*PCMR*) package in R version 3.1.4. Drugs were then grouped in ranked categories from the most active to the inactive. The distribution of median across drugs in the same rank was compared using either Mann-Whitney U-test or Kruskal-Wallis test. Statistical tests were interpreted using a 5% significance level.

## Results

### Inhibitory effect of the anti-parasitic drugs on the kinetic of the motility of *Loa loa* mfs

**Fig. 2:**
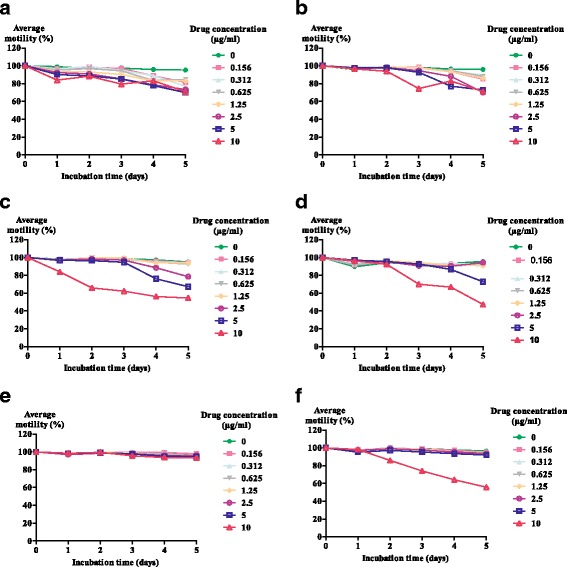
Kinetics of the motility of *L. loa* microfilariae exposed to different concentrations of anti-filarial, anti-schistosomal and anti-cancer drugs within 5 days of incubation. **a** Flubendazole (FLBZ). **b** Reduced flubendazole (RFLBZ). **c** Hydrolysed flubendazole (HFLBZ). **d** Ivermectin (IVM). **e** Praziquantel (PZQ). **f** Imatinib (Gleevec, GLV)

**Fig. 3:**
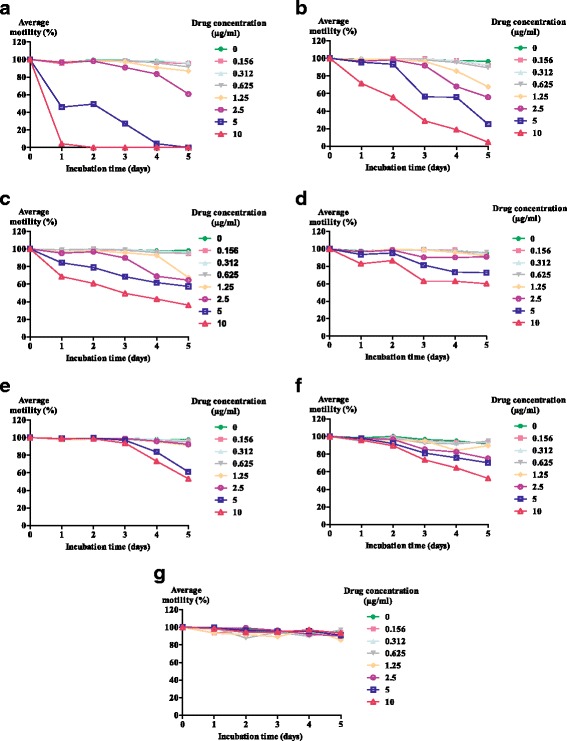
Kinetic of the motility of *L. loa* microfilariae exposed to different concentrations of antiprotozoal drugs within 5 days of incubation. **a** Mefloquine (MFQ). **b** Amodiaquine (AM). **c** Chloroquine (CQ). **d** Quinine (QN). **e** Artesunate (ATS). **f** SCYX-7158. **g** Fexinidazole

A total of 13 anti-parasitic drugs were screened against *L. loa* mf (Additional file 1: Table S1). The susceptibility of parasite motility to the drugs over time is shown in Figs. 2 and 3. Generally, mf motility in negative control wells remained high and close to 100% over the 5 days of culture.

Figure 2 shows the mf motility with incubation time after treatment with filaricidal, anti-schistosomal and anticancer drugs. In the presence of FLBZ and its two metabolites at concentrations between 1.56–10 μg/ml, mf motility decreased progressively with time. FLBZ (Fig. 2a) and its reduced metabolite (Fig. 2b) displayed little activity against *L. loa* mf at concentrations ≤ 10 μg/ml. The motility of mf decreased slightly from the first day after incubation and fluctuated around 70% within the 5 days. Even at 10 μg/ml, the motility of the parasites remained above 60%. However, the hydrolysed metabolite affected mf motility from the first day at 10 μg/ml (Fig. 2c), and there was a decrease in worm motility with time. At 5 and 2.5 μg/ml, mf motility started to decrease from the third day, and the extent of reduction was concentration-dependent. At concentrations < 2.5 μg/ml, mf motility was comparable to that of negative controls. Although reduction of mf motility was also observed from day 3 in the presence of 10 μg/ml IVM to 47% on day 5, the motility was still > 70% at concentrations ≤ 5 μg/ml (Fig. 2d). The anti-schistosomal PZQ had no effect on the motility of *L. loa* mf at 10 μg/ml (Fig. 2e). GLV reduced mf motility at 10 μg/ml from the first day of incubation to 55% by day 5 (Fig. 2f), and the reduction in motility was concentration-dependent.

The effects of antiprotozoal drugs on mf motility are illustrated in Fig. 3. The anti-malarial agents MFQ, AQ and CQ reduced the motility of *L. loa* mf after 48 h incubation at concentrations > 0.625 μg/ml (Fig. 3a, b and c, respectively). With AQ and MFQ, the reduction of *L. loa* mf motility was concentration-dependent, with MFQ being more active at higher concentrations. Reductions in mf motility were obtained with QN and ATS at 5 and 10 μg/ml (Fig. 3d and e, respectively); however; mf exposed to ATS showed no change in motility during the first 48 h.

SCYX-7158 showed significant (Spearman’s rho = -0.94285, *P* = 0.01667) concentration-dependent activity in reducing *L. loa* mf motility (Fig. 3f). At 12.5 μg/ml, which is close to the C_max_ values (10 μg/ml) found in mice after a dose of 25 mg/kg, the reduction in motility was approximately 50% after 5 days of incubation. Fexinidazole had no significant effect on mf motility at 10 μg/ml; all worms were actively motile (Fig. 3g).

### Relative reduction of mf motility and concentration reduction 50 (CR_50_)

**Fig. 4:**
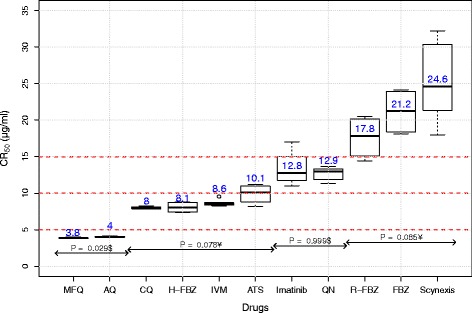
Comparative activity of the various drugs against *L. loa* microfilariae. Box-plots show the activities of the various drugs (CR_50_) against *L. loa* microfilariae. Fexinidazole and PZQ are not represented here because they were inactive. $: *P*-value for Mann-Whitney U-test. ¥: *P*-value for Kruskal-Wallis test

A summary of drug effects on mf motility of the mfs with respect to controls for the 3rd and 5th days in culture is shown in Table 1, and CR_50_ values are compared in Fig. 4. Drugs tested varied in their activity against mf. The relative reduction of mf motility exposed to MFQ at 10 μg/ml was 100% by day 3. The % reduction of mf motility after 5 days exposure to FLBZ, R-FLBZ and H-FLBZ at concentrations of 10 μg/ml was 25.3%, 25.8% and 42.8%, respectively. Three days post-exposure of mf to these drugs at 10 μg/ml reduced mf motility by 18.5%, 24.2 and 36.8%, respectively.

**Table 1:** Relative effects of the agents tested on mf motility at 3 and 5 days of culture at 1.25 and 10 μg/ml (% reduction from controls) and the CR_50_ values after 5 days incubation

Ranking	Drug	Motility reduction after 3 days^a^		Motility reduction (%) after 5 days^a^		Summary statistics for CR_50_				
		1.25 μg/ml	10 μg/ml	1.25 μg/ml	10 μg/ml	Mean ± SD	Median	Geometric mean	Interquartile range	95% CI
1 (Highly effective)	MFQ	1.8	100	9.3	100	3.9 ± 0.1	3.8	3.9	0.16	3.7–4.0
	AMQ	2.7	70.6	29.8	95.1	4.1 ± 0.1	4	4.1	0.1	4.0–4.1
2	CQ	3.7	10.3	31.3	63.3	8 ± 0.2	8	8	0.37	7.7–8.3
	H-FLBZ	7	36.8	0.9	42.5	8.1 ± 0.7	8.1	8	1.36	6.9–9.3
	IVM	2	24.4	5	50.6	8.7 ± 0.5	8.6	8.7	0.83	8.1–9.3
	ATS	0.3	5.6	6.5	45.4	9.9 ± 1.4	10.1	9.8	2.58	7.7–12.1
3	QN	0.4	36.4	2.7	36.5	12.7 ± 0.9	12.9	12.7	1.65	11.8–13.6
	GLV	0.5	25.2	1.3	42.1	13.4 ± 2.6	12.8	13.2	4.64	9.3–17.5
4	R-FLBZ	0.6	24.2	10.2	25.8	17.6 ± 3	17.8	17.5	5.56	12.9–22.4
	FLBZ	7.2	18.5	12.9	25.3	21.2 ± 3.2	21.2	21	5.79	16.1–26.3
	SCYX-7851	0.1	18.1	1	31.4	25.4 ± 5.6	24.6	24.9	10.64	20.3–30.6
5 (Not effective)	PZQ	0.2	3.1	2.1	3.5	–	–	–	–	–
	Fexinidazole	0.4	0.6	1.6	2.6	–	–	–	–	–

*Abbreviations*: *CI* confidence interval, *SD* standard deviation, – CR_50_ values for of PZQ and fexinidazole were not estimated, because they were inactive against *L. loa* mf

^a^% reduction relative to the negative control culture

The drugs that effected *Loa* mf in vitro most are MF and AMQ, while PZQ and fexinidazole displayed no activity. Based on CR_50_ values, the drugs tested were ranked in 5 categories in term of their effects (Table 1, Fig. 4). The first category includes the most active drugs with lowest CR_50_ values: MFQ and AM, with CR_50_ values of 3.9 ± 0.09 and 4.0 ± 0.06 μg/ml, respectively. The second group, with CR_50_ values between 8 and 10 μg/ml, includes CQ, H-FBZ, IVM and ATS, while the third group (12 < mean CR_50_ < 14 μg/ml) (if 10 μg/ml was the highest concentration tested, you cannot have a CR_50_ value above this) was the group of QN and GLV. The fourth group included drugs with very limited activity at 10 μg/ml (FLBZ and its reduced metabolite and SCYX-7851). The last group included the two inactive drugs, fexinidazole and PZQ, with undetermined CR_50_ values.

The Kruskal-Wallis chi-square value was 46.3636 (*df* = 10, *P* = 1.232e-06), indicating significant differences in the activities between various active drugs. Within a group of drugs with similar activities, differences were determined using Mann-Whitney U-test and Kruskal-Wallis test. Although MFQ and AQ were classified in the same group, MFQ had the significantly (*U* = 16, *P* = 0.02857) lowest value of CR_50_. However, no difference was found between the drugs within each of the remaining groups.

### Lethal effect of the anti-parasitic drugs on mf

**Fig. 5:**
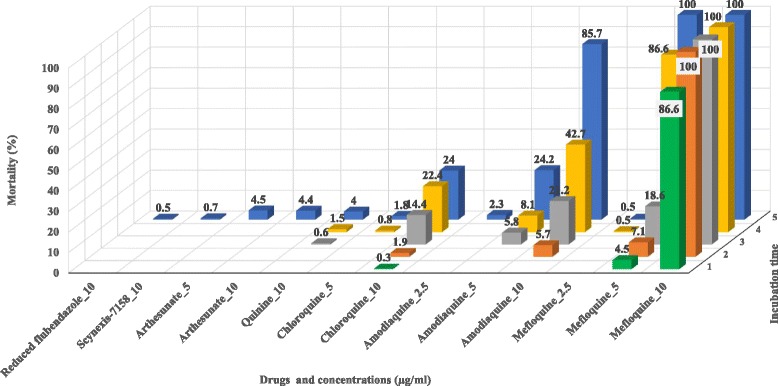
Mortality of *L. loa* microfilariae exposed to different concentrations of the active drugs. Drugs indicated here are those that killed at least one microfilaria at the concentration indicated

In addition to reduction in mf motility, lethal effects (score 0) were observed with some drugs (Fig. 5). This effect was more pronounced with MFQ than with AQ, given that at 10 μg/ml, 100% of mf were dead after 24 h (Fig. 5, Additional file 2: Table S2). Mortality rates for cultures exposed to MFQ increased with time and drug concentration. At 5 μg/ml MFQ the rates on day 1 and 5 were 4.5 and 100%, respectively, while for 2.5 μg/ml AQ, these rates were 2.3 and 85.7%, respectively. No immotile mf was found in wells containing FLBZ, H-FLBZ, fexinidazole, IVM or GLV at 10 μg/ml, the highest concentration tested, at the 5 day time point. On day 4, SCYX-7158 showed lethal effect on 0.7% of the *L. loa* mf at 10 μg/ml.

## Discussion

Post-treatment SAEs constitute a significant challenge for mass drug administration programmes in countries with endemic onchocerciasis and lymphatic filariasis and co-endemic loiasis. IVM and diethylcarbamazine, the drugs of choice for these diseases, can induce SAE in *Loa-*infected patients [4, 45]. As these SAEs are thought to be associated with the destruction of huge numbers of mf by these drugs, a principal aim of scientists and field program managers is to develop a way of reducing the circulating number of *Loa* mf without destroying a high number at once and thus avoid the SAEs. Development of such a drug requires investigation of the activity of selected candidates on *L. loa* mf using various experimental models. In this study, we assessed the predictive value of an in vitro system for the evaluation of drugs against loiasis.

FLBZ, a promising macrofilaricide candidate [19], was proposed to be useful in loiasis endemic areas based on the lack of activity against mf observed in various animal models [19]. Our results indicate that FLBZ at concentrations up to 10 μg/ml reduced the motility of *L. loa* mf progressively to a minor degree but did not kill the parasites during 5 days of culture. This indicates that the drug has relatively little direct effect on *L. loa* mf in vitro, and may not kill *L. loa* mf in vivo, as has been found with other filariae [21, 24]. The same finding was observed previously with *L. loa* and *B. malayi* mf exposed to FLBZ in vitro [25]. In this study, H-FLBZ was slightly more active against mf than FLBZ or R-FLBZ; however, it is generally thought that R-FLBZ but not H-FLBZ is a bioactive anthelmintic, with the latter present at only very low plasma levels. Our findings support previous results with other filarial species that showed that FLBZ had little or no effect on mf [21, 24]. In earlier studies in humans, no evidence of immediate mf destruction was found after i.m. injections of FLBZ [46], which was supported by a notably slow reduction of the population of *O. volvulus* mf over time; likewise, there is a lack of microfilaricidal activity of this drug on *B. pahangi* [21]. FLBZ nevertheless appears to have significant effects on adult filariae, and in all likelihood considerably more than IVM does. Thus, its ability to eliminate adult worms [20–23, 47] without a potentially dangerous direct effect on mf makes it a good candidate for use in the global elimination/eradication programs for filarial infections.

In vitro studies are useful in providing information concerning the direct effect of drugs against organisms such as filarial parasites. However, many antiparasitic drugs require a contribution from the host to be effective, components that are typically missing from in vitro assays; this indeed appears to be the case with IVM [15, 16]. It is important, therefore, to compare in vitro results with results from in vivo models, and to include both positive and negative controls in any in vitro study. In this study, we included a range of chemotherapeutic agents that have previously been tested against filariae in vitro. Our findings are consistent in general with expectations from previous in vitro and in vivo work and indicate that our culture system over a period of five days produces comparable and valid data. Among the agents used for comparison here, the anti-malarials ATS, CQ, AQ and QN have all been found inactive against *L. loa* mf in humans [48]. Except CQ, whose in vivo microfilaricidal activity has been proven against *O. volvulus* [49], agents that are known to be active against filariae and that were also active in our present studies are MQ [25] and imatinib, an anti-cancer compound recently added to the list of active filaricidal drugs [17, 18, 50]. PZQ, an anti-schistosomal drug, was also tested but was inactive in this system.

HAT has considerable public health and economic impacts and is usually fatal when untreated. Current drugs suffer from poor safety profiles, inadequate regimens, limited effectiveness, and high cost. In an effort to develop alternative therapies, two new candidates, fexinidazole and SCYX-7158, were selected to enter preclinical studies on the basis of their safety and potential to treat advanced-stages of sleeping sickness. Most of the foci of sleeping sickness situated in the forest zones of East and Central Africa were identified as loiasis endemic areas, constituting an obstacle to onchocerciasis control and lymphatic filariasis elimination in sub-Saharan Africa due to SAEs resulting from massive killing of *L. loa* mf by IVM. It is important to determine if similar SAEs are as threat to the use of these new trypanocides.

Studies on *L. loa*-trypanosome co-infections have not been as extended as those on the co-occurrence of *L. loa* and *Onchocerca* spp. The heterogeneous nature of the associated clinical manifestations poses problems for prevention and timely patient management [51]. It is therefore obvious that any drug discovery strategy for HAT should take into consideration the issue of SAEs in co-infection with *L. loa*. To reduce a risk of SAEs, any drug to be massively used to control a disease in *Loa* endemic area, should have little or no effect on *L. loa* microfilaria.

Although fexinidazole and SCYX-7158 showed promising pharmacological activity and safety profiles in pre-clinical studies, their risk in inducing SAEs is not yet predicted. The present work thus evaluated fexinidazole and SCYX-7158 against *L. loa* mf in vitro. Fexinidazole had no significant effect on the viability of *L. loa* mf during 5 days of incubation. This highlighted the specificity of the drug to trypanosomes. In vitro trypanocidal activity of fexinidazole has already been investigated with IC_50_ range (0.2–0.9 μg/ml) against *T. brucei* species [35]. At a concentration close to 50 times these IC_50_ values, the drug had no lethal or paralyzing effect on *L. loa* mf, suggesting that fexinidazole is compatible with MDA programme in *Loa* endemic areas. However, we cannot rely only on these data to predict the safety of fexinidazole in areas of loaisis endemicity, because it is known that even though IVM induced SAEs by killing massive numbers of *L. loa* mf, it lacks direct microfilaricidal activity in vitro [15]. Therefore, it will be interesting to verify the specific activity of the drug in a murine model of loiasis and trypanosomiasis co-infection. Although the murine model of trypanosomiasis is already available, this is not a case with *L. loa* mf. However, there is an ongoing effort to develop a mouse model of loiasis.

SCYX-7158 exhibited little activity on *L. loa* mf. At concentrations between 12.5–50 μg/ml, this drug inhibited the motility of *L. loa* mf by more than 50% by day 5. In addition, on days 4 and 5, close to 3% of *L. loa* mf were killed. However, these lethal concentrations were higher than plasma C_max_ levels (10 μg/ml) previously found in mice after a 25 mg/kg dose [36]. As discussed above, an in vivo system is required to validate the non-selectivity of this drug, and predict it risk of SAEs in loiasis co-endemic area.

## Conclusions

This study reported, to our knowledge for the first time, the in vitro microfilaricidal activities of antimalarial drugs MFQ AQ, CQ, ATS and QN against *L. loa* mf. These findings highlight the need to investigate the effect of antimalarial therapy on loiasis microfilaraemia in the endemic areas. IVM, GLV, FLBZ and its major metabolites had little effect on *L. loa* mf in vitro. PZQ was inactive against *L. loa* mf in vitro. Fexinidazole and SCYX-7158 showed no and little effect, respectively, on *L. loa* mf viability at concentrations close to C_max_ values. However, further studies need to be done to confirm these findings in an in vivo model. These drugs remain candidates for clinical trials for sleeping sickness, even in areas of trypanosomiasis and loiasis co-endemicity. Given that one cannot rely on in vitro activity to predict the effects of a drug because of pharmacokinetic and pharmacodynamic properties that are not incorporated in culture, it is advised to assess the effect of the above drugs in an in vivo model of loiasis. The selectivity of the drug on trypanosomiasis *vs* loiasis can be assessed in an experimental model such as the recent development of patent infections of *L. loa* in a murine model.

## Additional files

Additional file 1: **Table S1.** Effects of different drug concentrations on the motility of *L. loa* mf. (DOCX 33 kb)

Additional file 2: **Table S2.** Average rate of *Loa* mf mortality (%) after exposure to different drug concentrations at different time intervals. (DOCX 20 kb)

## Abbreviations

AQ: Amodiaquine
ATS: Artesunate
CCM: Complete culture medium
CQ: Chloroquine
CR_50_: Concentration reduction 50
DMSO: Dimethyl sulfoxide
DNDi: Drug for Neglected Diseases initiative
FLBZ: Flubendazole
GLV: Gleevec (imatinib)
H-FLBZ: Hydrolyzed flubendazole
IC_50_: Inhibitory concentration 50
IVM: Ivermectin
Mf: Microfilaria
MFQ: Mefloquine
MTT: 3-(4,5-dimethylthiazol-2-yl)-2,5-diphenyltetrazolium bromide
PZQ: Praziquantel
QN: Quinine
R-FLBZ: Reduced flubendazole
SCYX-7158: Scynexis-7158
