# Supplementary material for: Heterogeneity in the in vitro susceptibility of Loa loa microfilariae to drugs commonly used in parasitological infections
Source: Parasit Vectors. 2018 Apr 4;11:223. doi: 10.1186/s13071-018-2799-3 (PMC5883330; doi:10.1186/s13071-018-2799-3)
Supplement: Supplementary file 1 — Table S1. Effects of different drug concentrations on the motility of L. loa mf. (DOCX 33 kb) [file 13071_2018_2799_MOESM1_ESM.docx]

**Additional file** **1: Table S1.** Effects of different drug concentrations on the motility of *L. loa* mf

| Drugs | Concentration (µg/mL) | Incubation time (days) | | | | | |
| --- | --- | --- | --- | --- | --- | --- | --- |
|  |  | 0 | 1 | 2 | 3 | 4 | 5 |
| Control (0.1% DMSO) | | 100±0 | 99.70±0.88 | 98.35±2.05 | 98.51±2.53 | 97.37±2.68 | 94.82±6.00 |
| AQ | 0 | 100±0 | 99.07±1.26 | 98.99±1.33 | 99.10±0.75 | 97.65±1.99 | 96.52±2.61 |
|  | 0.156 | 100±0 | 97.39±0.82 | 99.42±0.66 | 99.39±0.72 | 97.29±0.78 | 91.84±3.16 |
|  | 0.312 | 100±0 | 96.67±1.20 | 99.17±0.68 | 99.17±0.68 | 97.59±1.46 | 92.30±3.26 |
|  | 0.625 | 100±0 | 97.88±1.75 | 98.05±0.91 | 98.10±1.76 | 95.31±2.44 | 89.23±4.32 |
|  | 1.25 | 100±0 | 98.69±1.52 | 98.61±1.37 | 96.43±1.80 | 85.57±5.58 | 67.75±4.17 |
|  | 2.5 | 100±0 | 96.64±1.82 | 98.44±1.83 | 91.80±2.53 | 68.00±2.31 | 55.82±3.22 |
|  | 5 | 100±0 | 95.51±1.54 | 93.12±3.21 | 56.31±2.54 | 55.88±2.69 | 25.26±1.48 |
|  | 10 | 100±0 | 71.56±2.97 | 55.73±1.74 | 29.10±2.98 | 19.09±3.37 | 4.75±1.88 |
| ATS | 0 | 100±0 | 98.75±0.84 | 99.29±1.28 | 99.30±0.55 | 97.82±2.36 | 97.67±2.31 |
|  | 0.156 | 100±0 | 98.15±1.39 | 99.16±0.66 | 98.88±0.72 | 95.64±1.71 | 96.49±2.30 |
|  | 0.312 | 100±0 | 97.52±1.06 | 98.62±1.16 | 98.83±1.36 | 98.49±0.98 | 96.52±1.71 |
|  | 0.625 | 100±0 | 98.00±1.62 | 98.50±1.76 | 98.13±1.61 | 95.27±0.91 | 95.43±2.78 |
|  | 1.25 | 100±0 | 97.47±0.92 | 98.14±0.65 | 99.03±0.77 | 95.17±1.62 | 91.29±2.65 |
|  | 2.5 | 100±0 | 98.47±1.04 | 98.98±0.87 | 98.98±0.87 | 95.90±1.19 | 92.40±2.16 |
|  | 5 | 100±0 | 98.84±1.13 | 99.35±0.73 | 96.95±1.01 | 83.63±5.11 | 61.05±4.21 |
|  | 10 | 100±0 | 98.82±1.94 | 98.54±1.26 | 93.72±2.76 | 73.14±5.51 | 53.29±8.54 |
| CQ | 0 | 100±0 | 99.02±1.31 | 99.31±1.39 | 99.54±0.93 | 98.03±2.28 | 98.38±2.02 |
|  | 0.1 | 100±0 | 100±0 | 97.73±2.69 | 98.73±2.29 | 95.62±3.39 | 92.78±4.90 |
|  | 0.156 | 100±0 | 97.08±0.32 | 99.39±0.71 | 98.40±1.17 | 96.41±0.49 | 95.03±1.40 |
|  | 0.312 | 100±0 | 96.80±2.11 | 99.51±0.57 | 99.73±0.54 | 97.31±1.36 | 96.61±1.16 |
|  | 0.625 | 100±0 | 99.12±0.59 | 100±0 | 98.98±1.25 | 95.79±2.02 | 94.77±0.87 |
|  | 1.25 | 100±0 | 96.12±1.11 | 98.00±1.46 | 95.87±1.89 | 92.60±0.90 | 67.59±1.26 |
|  | 2.5 | 100±0 | 95.25±0.72 | 96.84±0.73 | 89.85±1.40 | 68.98±2.09 | 64.52±1.45 |
|  | 5 | 100±0 | 84.33±4.32 | 79.04±2.38 | 68.51±2.80 | 61.81±1.25 | 57.46±1.47 |
|  | 10 | 100±0 | 68.69±4.72 | 60.91±5.93 | 49.51±11.14 | 43.10±13.93 | 36.10±8.84 |
| FLBZ | 0 | 100±0 | 99.02±0.95 | 96.88±1.92 | 97.45±1.75 | 95.81±1.12 | 95.18±1.38 |
|  | 0.1 | 100±0 | 87.26±8.34 | 98.77±1.92 | 88.03±7.46 | 89.89±4.20 | 79.22±7.66 |
|  | 0.156 | 100±0 | 97.07±0.98 | 98.41±2.24 | 97.05±1.65 | 88.72±4.16 | 81.36±3.93 |
|  | 0.312 | 100±0 | 96.57±0.57 | 99.33±0.77 | 94.94±1.30 | 88.84±2.40 | 76.93±7.09 |
|  | 0.625 | 100±0 | 95.20±2.93 | 96.94±1.49 | 94.38±2.91 | 83.49±7.56 | 84.66±3.82 |
|  | 1.25 | 100±0 | 93.51±1.92 | 93.07±2.28 | 90.42±2.35 | 83.27±5.14 | 82.94±3.89 |
|  | 2.5 | 100±0 | 92.37±1.90 | 90.92±1.92 | 85.37±5.55 | 78.68±3.75 | 73.21±3.52 |
|  | 5 | 100±0 | 90.20±5.58 | 88.64±6.08 | 85.23±4.00 | 77.82±3.75 | 69.91±2.22 |
|  | 10 | 100±0 | 83.95±11.84 | 88.47±5.47 | 79.46±7.71 | 83.14±7.30 | 71.10±6.10 |
| H-FLBZ | 0 | 100±0 | 98.29±1.50 | 98.65±0.82 | 98.73±1.36 | 97.41±1.52 | 95.06±1.75 |
|  | 0.1 | 100±0 | 99.71±0.69 | 99.31±1.07 | 98.66±2.20 | 98.15±1.61 | 91.30±5.54 |
|  | 0.156 | 100±0 | 97.22±1.23 | 97.59±0.71 | 97.59±0.71 | 96.23±1.39 | 94.47±3.05 |
|  | 0.312 | 100±0 | 97.47±0.99 | 99.71±0.57 | 97.46±1.56 | 95.13±1.04 | 93.93±3.24 |
|  | 0.625 | 100±0 | 98.15±1.24 | 99.69±0.62 | 98.80±0.97 | 94.70±2.16 | 92.61±6.69 |
|  | 1.25 | 100±0 | 96.05±1.30 | 100±0 | 99.64±0.72 | 93.99±2.23 | 94.23±2.07 |
|  | 2.5 | 100±0 | 97.09±0.70 | 98.49±2.25 | 97.06±2.45 | 88.50±5.79 | 78.53±9.97 |
|  | 5 | 100±0 | 96.98±0.97 | 96.52±2.03 | 94.71±1.92 | 76.29±5.50 | 67.25±5.33 |
|  | 10 | 100±0 | 83.95±10.29 | 65.94±17.20 | 62.37±3.60 | 56.36±8.03 | 54.68±11.09 |
| GLV | 0 | 100±0 | 97.04±0.82 | 98.90±1.27 | 99.16±1.03 | 97.64±0.82 | 96.51±1.66 |
|  | 0.1 | 100±0 | 100±0 | 99.49±0.94 | 97.70±3.00 | 93.15±6.79 | 95.55±2.26 |
|  | 0.156 | 100±0 | 97.31±0.99 | 99.29±0.86 | 99.05±1.26 | 97.21±1.79 | 95.26±1.12 |
|  | 0.312 | 100±0 | 97.01±1.13 | 98.43±2.36 | 97.28±3.93 | 94.89±1.20 | 94.60±1.18 |
|  | 0.625 | 100±0 | 97.71±1.30 | 100±0 | 98.50±1.74 | 95.34±1.93 | 95.31±1.21 |
|  | 1.25 | 100±0 | 97.46±2.29 | 99.34±0.76 | 98.65±1.97 | 95.59±1.53 | 95.28±1.78 |
|  | 2.5 | 100±0 | 97.26±0.80 | 99.02±0.79 | 97.73±1.22 | 95.29±1.09 | 93.75±1.83 |
|  | 5 | 100±0 | 95.27±2.18 | 97.23±1.64 | 95.37±2.26 | 93.44±2.36 | 92.00±3.41 |
|  | 10 | 100±0 | 98.26±2.90 | 85.96±7.54 | 74.18±10.59 | 64.19±4.49 | 55.89±4.71 |
| IVM | 0 | 100±0 | 89.68±2.88 | 94.22±3.04 | 92.81±2.23 | 93.34±2.05 | 95.76±3.11 |
|  | 0.312 | 100±0 | 93.01±3.49 | 95.78±2.77 | 92.43±4.11 | 93.62±3.45 | 91.62±3.30 |
|  | 0.625 | 100±0 | 91.91±3.57 | 95.71±3.87 | 92.82±3.93 | 90.84±4.36 | 91.49±4.59 |
|  | 1.25 | 100±0 | 96.11±2.62 | 96.66±2.13 | 94.38±4.74 | 91.63±3.99 | 90.94±4.45 |
|  | 2.5 | 100±0 | 95.61±3.02 | 95.48±1.11 | 90.66±5.52 | 90.45±5.25 | 94.37±1.69 |
|  | 5 | 100±0 | 97.02±2.98 | 95.52±5.37 | 92.65±4.50 | 86.79±6.01 | 72.82±3.59 |
|  | 10 | 100±0 | 96.39±3.54 | 92.37±7.25 | 70.16±5.32 | 66.85±0.42 | 47.34±10.46 |
| MFQ | 0 | 100±0 | 96.96±0.83 | 99.66±0.53 | 99.26±0.85 | 98.26±1.73 | 95.96±1.74 |
|  | 0.156 | 100±0 | 95.32±1.16 | 99.04±0.82 | 98.16±2.02 | 96.63±2.92 | 95.41±1.80 |
|  | 0.312 | 100±0 | 97.29±1.43 | 97.88±1.17 | 98.30±1.31 | 99.06±1.14 | 95.91±2.63 |
|  | 0.625 | 100±0 | 97.42±0.74 | 98.84±0.77 | 99.11±0.72 | 96.11±1.17 | 91.67±1.96 |
|  | 1.25 | 100±0 | 97.64±0.50 | 98.40±1.40 | 97.45±1.30 | 90.95±3.64 | 87.06±4.62 |
|  | 2.5 | 100±0 | 96.87±0.94 | 98.12±1.45 | 90.95±3.33 | 83.59±7.16 | 60.82±3.38 |
|  | 5 | 100±0 | 45.93±17.21 | 49.40±11.69 | 27.14±3.32 | 4.44±1.63 | 0 |
|  | 10 | 100±0 | 4.47±1.15 | 0 | 0 | 0 | 0 |
| PZQ | 0 | 100±0 | 98.37±0.69 | 99.68±0.64 | 98.70±1.09 | 98.05±0.77 | 96.75±1.73 |
|  | 0.156 | 100±0 | 98.59±1.02 | 99.40±0.70 | 99.40±0.70 | 99.40±0.70 | 97.99±1.41 |
|  | 0.312 | 100±0 | 97.56±2.29 | 99.33±1.33 | 98.71±1.10 | 97.09±1.32 | 96.03±1.94 |
|  | 0.625 | 100±0 | 97.60±0.99 | 99.19±0.56 | 98.82±1.05 | 97.69±1.69 | 95.92±1.08 |
|  | 1.25 | 100±0 | 98.13±0.37 | 99.77±0.45 | 98.96±0.91 | 95.38±1.49 | 94.76±1.96 |
|  | 2.5 | 100±0 | 97.05±1.19 | 98.65±1.12 | 98.03±0.74 | 95.91±1.83 | 95.91±1.83 |
|  | 5 | 100±0 | 98.24±2.07 | 99.09±0.62 | 97.88±0.86 | 95.02±0.82 | 94.65±0.91 |
|  | 10 | 100±0 | 98.67±1.02 | 99.68±0.64 | 95.69±1.72 | 93.76±2.55 | 93.37±1.97 |
| QN | 0 | 100±0 | 97.81±0.99 | 98.77±1.23 | 99.33±0.77 | 98.38±1.33 | 94.90±2.12 |
|  | 0.156 | 100±0 | 96.65±0.99 | 98.99±0.98 | 98.94±0.92 | 98.82±1.52 | 91.23±2.14 |
|  | 0.312 | 100±0 | 96.87±0.95 | 99.24±0.89 | 98.92±1.30 | 98.25±2.19 | 94.15±1.80 |
|  | 0.625 | 100±0 | 96.85±1.15 | 98.32±1.35 | 99.38±0.71 | 97.36±2.57 | 96.18±1.70 |
|  | 1.25 | 100±0 | 96.91±0.57 | 99.64±0.88 | 98.91±0.90 | 96.02±2.20 | 92.31±4.73 |
|  | 2.5 | 100±0 | 96.54±0.94 | 98.75±1.38 | 90.40±2.33 | 90.26±1.33 | 91.00±2.98 |
|  | 5 | 100±0 | 93.65±2.66 | 95.31±2.69 | 81.51±8.06 | 73.41±2.72 | 72.60±2.93 |
|  | 10 | 100±0 | 83.19±3.27 | 86.78±5.84 | 63.17±1.38 | 62.99±1.59 | 60.24±2.19 |
| R-FLBZ | 0 | 100±0 | 95.68±3.06 | 98.42±1.34 | 98.26±1.10 | 96.48±1.61 | 95.92±1.32 |
|  | 0.1 | 100±0 | 98.20±3.40 | 97.80±2.51 | 91.50±6.54 | 90.69±3.99 | 82.43±7.28 |
|  | 0.156 | 100±0 | 97.36±0.30 | 97.59±1.85 | 98.44±1.42 | 93.43±2.45 | 85.19±4.34 |
|  | 0.312 | 100±0 | 97.80±0.57 | 98.32±1.29 | 97.51±0.86 | 93.75±2.38 | 89.85±3.57 |
|  | 0.625 | 100±0 | 98.24±0.52 | 98.99±0.70 | 97.45±1.56 | 94.55±1.30 | 88.11±1.36 |
|  | 1.25 | 100±0 | 96.25±1.05 | 99.17±1.67 | 97.65±1.06 | 93.36±0.81 | 86.11±1.93 |
|  | 2.5 | 100±0 | 97.18±2.07 | 98.08±0.83 | 94.51±4.71 | 88.25±2.46 | 70.13±4.61 |
|  | 5 | 100±0 | 97.74±0.83 | 97.74±0.83 | 92.69±0.90 | 77.27±2.53 | 72.94±1.80 |
|  | 10 | 100±0 | 96.66±6.40 | 93.84±4.51 | 74.48±7.68 | 82.93±7.86 | 71.16±4.19 |
| Fexinidazole | 0 | 100±0 | 99.15±4.44 | 98.13±4.33 | 95.09±3.63 | 92.68±6.91 | 94.73±5.77 |
|  | 0.625 | 100±0 | 100±0 | 87.62±4.84 | 94.44±11.31 | 89.71±6.8 | 97.22±15.31 |
|  | 1.25 | 100±0 | 93.39±7.2 | 91.67±8.34 | 89.25±0.35 | 97.73±11.54 | 85.79±2.5 |
|  | 2.5 | 100±0 | 98.75±7.22 | 99.22±10.51 | 96.57±2.65 | 92.16±7.64 | 90.12±7.13 |
|  | 5 | 100±0 | 99.63±1.07 | 96.33±6.54 | 95.07±1.61 | 95.86±2.57 | 91.08±8.13 |
|  | 10 | 100±0 | 98.52±6.66 | 94.2±8.18 | 94.88±4.65 | 97.01±20.74 | 93.5±8.66 |
| Scynexis-7158 | 0 | 100±0 | 98.64±2.05 | 100±0 | 96.6±4.29 | 95.12±2.18 | 91.56±6.15 |
|  | 0.625 | 100±0 | 94.89±2.9 | 98.17±1.66 | 92.51±1.04 | 91.54±3.19 | 94.83±4.21 |
|  | 1.25 | 100±0 | 95.82±0.83 | 95.94±3.53 | 94.93±4.9 | 84.36±10.37 | 89.56±6.5 |
|  | 2.5 | 100±0 | 98.03±1.51 | 96.67±1.88 | 85.22±2.67 | 82.49±2.49 | 74.91±2.25 |
|  | 5 | 100±0 | 97.82±0.18 | 92.07±7.87 | 81.3±4.5 | 75.97±4.53 | 70.18±3.89 |
|  | 10 | 100±0 | 95.84±8.79 | 89.62±3.7 | 73.47±5.39 | 64.32±12.9 | 52.41±19.35 |
