# Supplementary material for: Heterogeneity in the in vitro susceptibility of Loa loa microfilariae to drugs commonly used in parasitological infections
Source: Parasit Vectors. 2018 Apr 4;11:223. doi: 10.1186/s13071-018-2799-3 (PMC5883330; doi:10.1186/s13071-018-2799-3)
Supplement: Supplementary file 2 — Table S2. Average rate of Loa mf mortality (%) after exposure to different drug concentrations at different time intervals. (DOCX 20 kb) [file 13071_2018_2799_MOESM2_ESM.docx]

**Additional file 2: Table S2.** Average rate of *Loa* mf mortality (%) after exposure to different drug concentrations at different time intervals

| Drugs | Drug concentration* (µg/mL) | Incubation time (days) | | | | |
| --- | --- | --- | --- | --- | --- | --- |
|  |  | 1 | 2 | 3 | 4 | 5 |
| DMSO (0.1%) | Negative control | 0 | 0 | 0 | 0 | 0 |
| FLBZ | 10 | 0 | 0 | 0 | 0 | 0 |
| R-FLBZ | 10 | 0 | 0 | 0 | 0 | 0.45 ± 1.78 |
| H-FLBZ | 10 | 0 | 0 | 0 | 0 | 0 |
| AQ | 2.5 | 0 | 0 | 0 | 0 | 2.27 ± 2.49 |
|  | 5 | 0 | 0 | 5.79 ± 3.39 | 8.05 ± 3.19 | 24.23 ± 4.44 |
|  | 10 | 0 | 5.67 ± 2.45 | 21.23 ± 4.78 | 42.73 ± 10.12 | 85.74 ± 5.64 |
| ATS | 5 | 0 | 0 | 0 | 0 | 4.52 ± 2.35 |
|  | 10 | 0 | 0 | 0 | 0 | 4.39 ± 5.57 |
| CQ | 5 | 0 | 0 | 0 | 0.83 ± 1.66 | 1.79 ± 2.08 |
|  | 10 | 0.31 ± 1.25 | 1.92 ± 3 | 14.44 ± 14.46 | 22.41 ± 20.69 | 23.96 ± 10.69 |
| MFQ | 2.5 | 0 | 0 | 0 | 0.46 ± 1.13 | 0.46 ± 1.13 |
|  | 5 | 4.54 ± 5.08 | 7.1 ± 8.63 | 18.57 ± 9.96 | 86.67 ± 4.89 | 100 |
|  | 10 | 86.6 ± 3.46 | 100 | 100 | 100 | 100 |
| QN | 10 | 0.64 ± 1.57 | 0 | 0.64 ± 1.57 | 1.52 ± 2.39 | 3.98 ± 2.83 |
| Scynexis-7158 | 10 | 0 | 0 | 0 | 0 | 0.7±2.0 |
| Fexinidazole | 10 | 0 | 0 | 0 | 0 | 0 |
| Imatinib | 10 | 0 | 0 | 0 | 0 | 0 |
| PZQ | 10 | 0 | 0 | 0 | 0 | 0 |
| IVM | 10 | 0 | 0 | 0 | 0 | 0 |

*: No dead mf was observed (mortality= 0 %) within five days at the drug concentrations below those indicated here.
